# Supplementary material for: Robust generation of transgenic mice by simple hypotonic solution mediated delivery of transgene in testicular germ cells
Source: Mol Ther Methods Clin Dev. 2016 Dec 7;3:16076–. doi: 10.1038/mtm.2016.76 (PMC5142465; doi:10.1038/mtm.2016.76)
Supplement: Supplementary Figures and Tables [file mtm201676-s1.pdf]

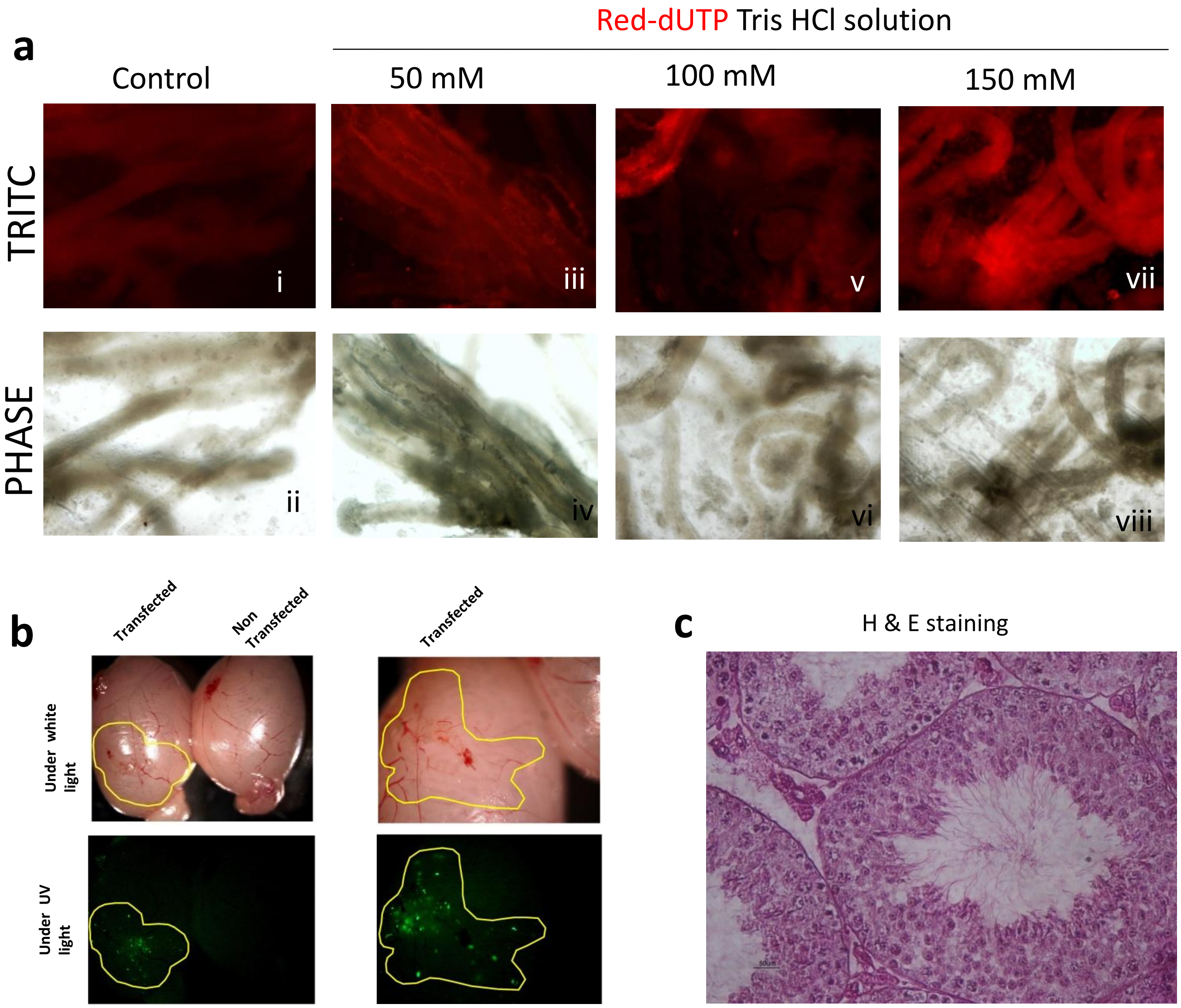

Figure S1. Hypotonic solution mediated transfection in testis

a) Transfection of Red dUTP with various concentrations of hypotonic Tris-HCl solution (i, ii) Control (non-transfected), (iii, iv) 50 mM, (v, vi) 100 mM, and (vii, viii) 150mM. Upper panel shows the images under UV (TRITC-filter) and lower panel shows the phase contrast images.

b) Expression of EGFP in transfected testis with pCX-Egfp construct suspended in 150 mM Tris-HCl solution, compared with non transfected testis.

c) Testis cross section post transfection with hypotonic solution (Tris HCl) injection. H & E = Hematoxylin and eosin.

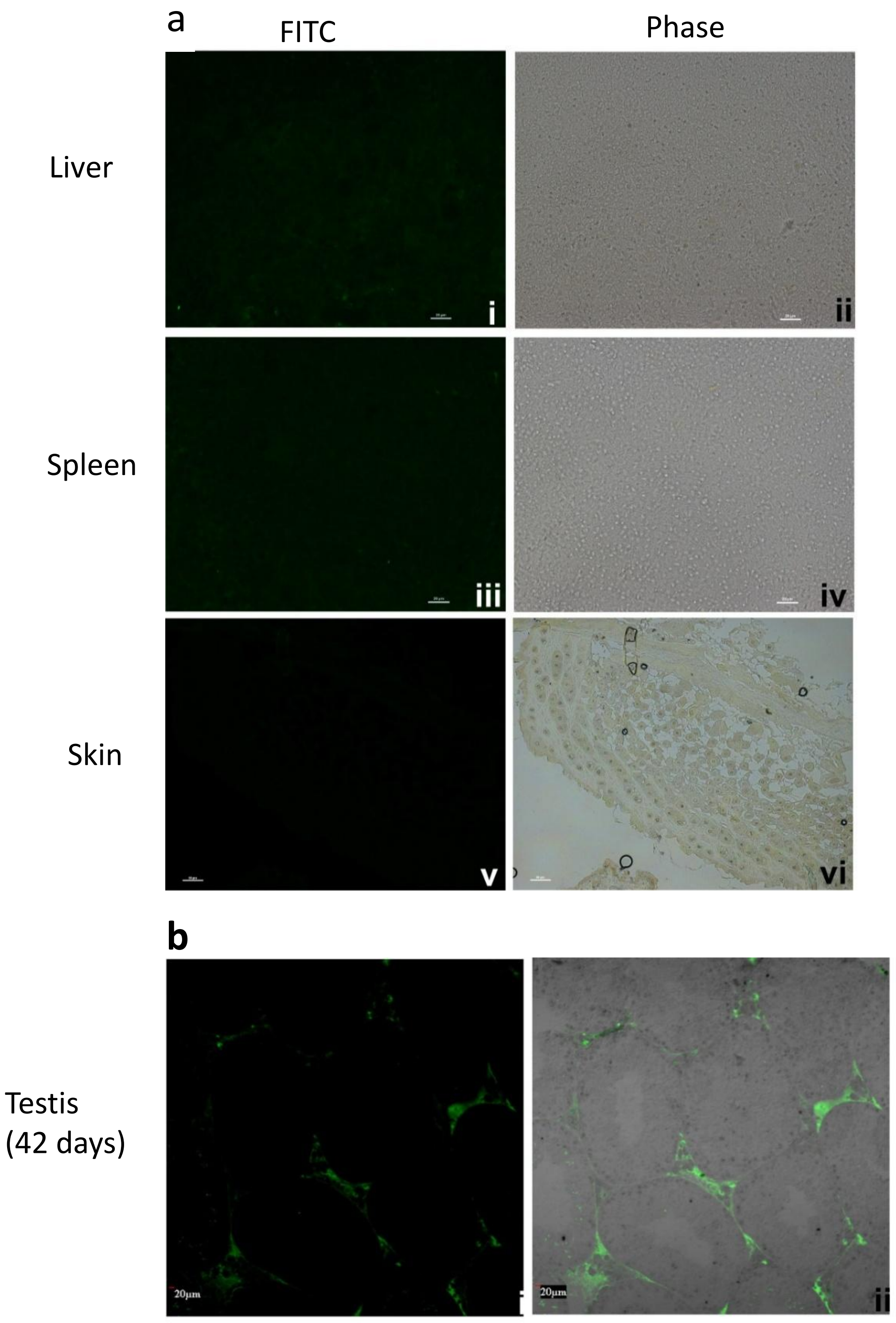

Figure S2. Expression of EGFP in *Amh-Ires2-Egfp* transgenic mouse
(a) Absence of EGFP expression in Liver (**i & ii)** ; Spleen **(iii & iv)** ; and skin **(v & vi)** of 5 days old *Amh-Ires2-Egfp* transgenic mouse. Scale bar: i - iv 20 µm; v & vi 50 µm.
(b) EGFP expression in testicular section of adult (42 days old) *Amh-Ires2-Egfp* transgenic mouse. Note: non-specific staining in interstitial area. Scale bar: 50 µm.

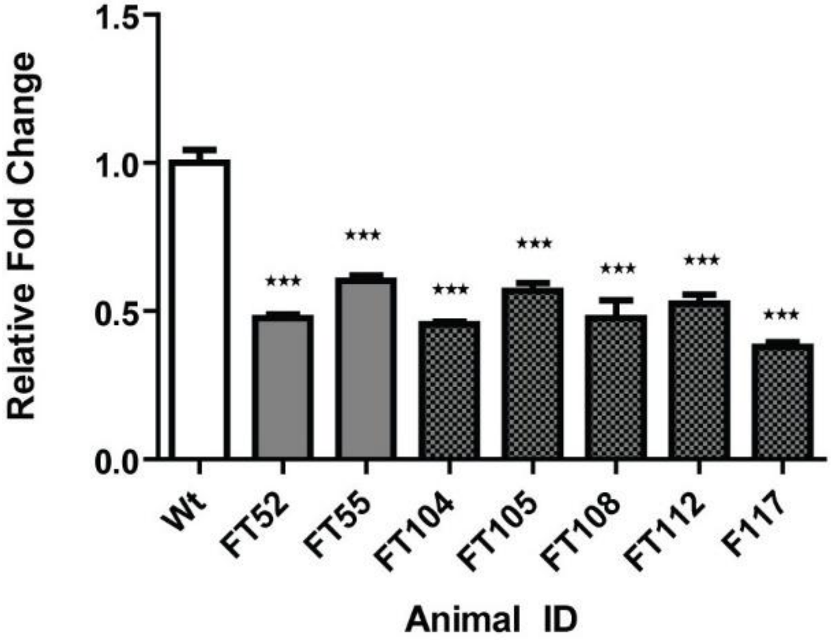

Figure S3. Expression of Fetuin-A in G2 generation of Fetuin-A knockdown transgenic mice

Relative fold changes in Fetuin-A mRNA expression of transgenic animals (G2 generations) relative to wild type animals. wt =wild type mice, FT - denotes *Fetuin- A* shRNA expressing transgenic mice. FT#represents seven different transgenic animals. Each bar generated from n=3 qRTPCR of same sample, represented as mean ± SEM. *** P<0.001.

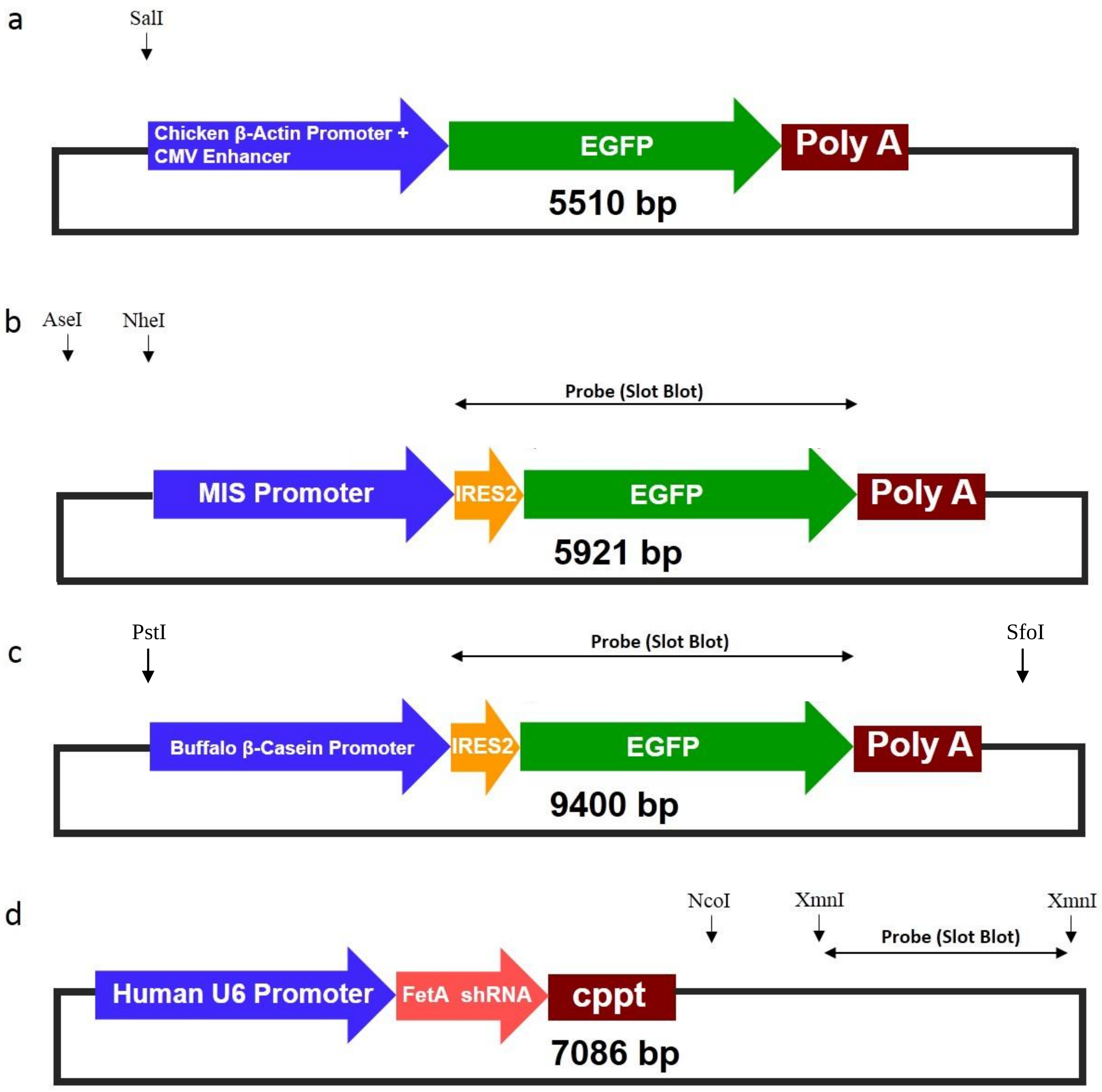

Figure S4. Construct details
(a) Vector map of *pCX-EGFP* vector construct
(b) Vector map of *Amh-Ires2-Egfp* vector construct.
(c) Vector Map of *Bucsn2-Ires2-Egfp* Vector Construct
(d) Vector map of *Fetuin-A* –shRNA vector construct

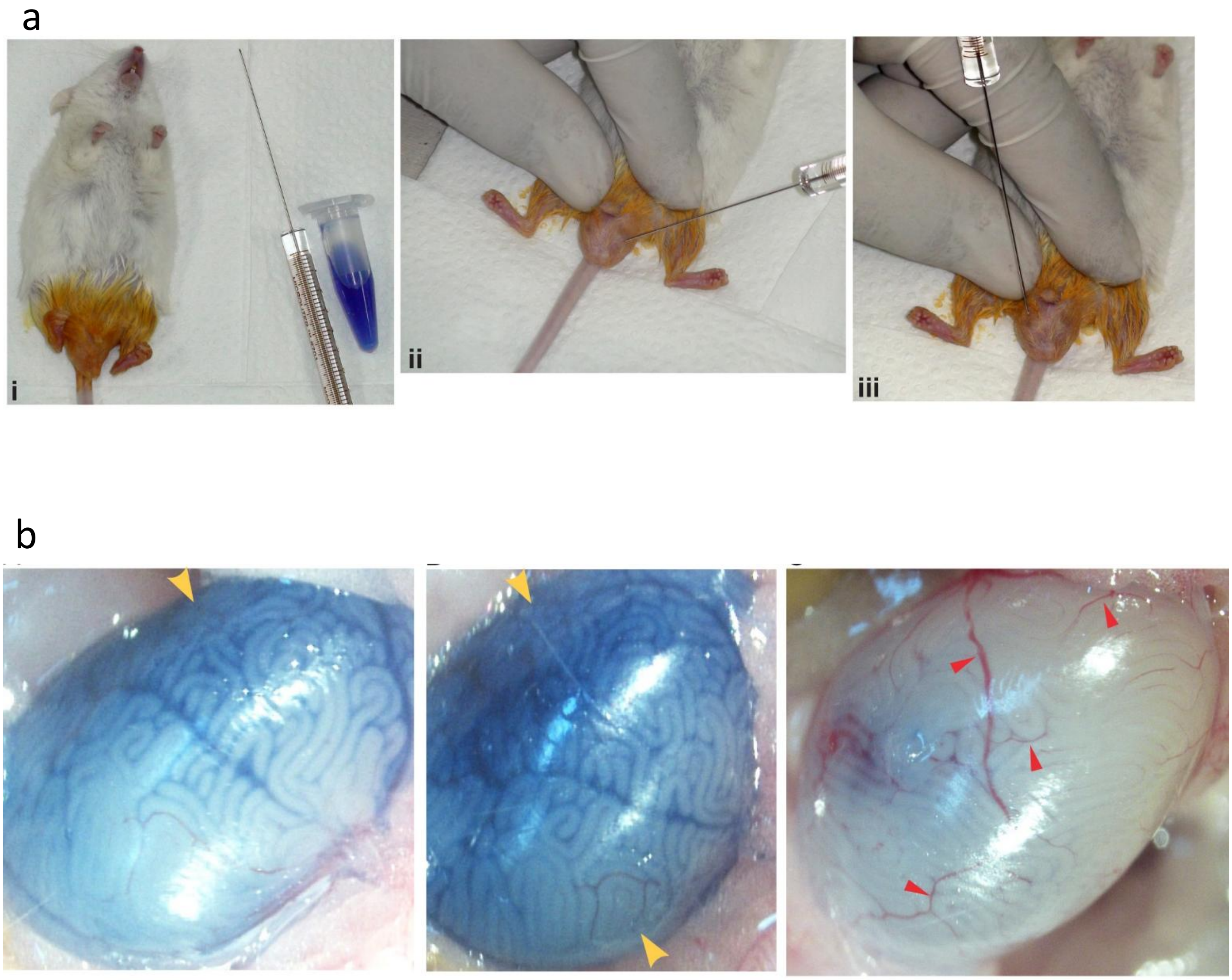

Figure S5. Injection of Hypotonic solution in testis

a) Procedure for injection of pDNA in the testis of mice.

b) Testis after injection. Yellow arrowhead shows diagonally opposite injection site. Red arrow head shows the blood vessels in testis.

Table S1. DNA concentration and conditions for standardizing *in vivo* testicular transfection using linearized pCX-EGFP plasmid suspended in Tris-HCl solution.

**Condition of Injection**	**Age on DOI (days)**	**DNA parameters**			**Injection parameters**	**Conc. of Tris-HCl (mM)**	**EGFP Fluoroscence**
		**Conc. (μg/μl)**	**Vol./ Testis (μl)**	**Amt. (μg)**	**No. of injection sites**		
EX 1	30±2	0.5	20	10	1	20	
EX 2	30±2	0.5	20	10	1	40	
EX 3	30±2	0.5	20	10	1	60	
EX 4	30±2	0.5	25	12.5	2	80	
EX 5	30±2	1.0	30	30	2	80	
EX 6	30±2	0.5	25	12.5	2	100	+
EX 7	30±2	1.0	25	25	2	100	
EX 8	30±2	0.5	25	12.5	2	125	++
EX 9	30±2	1.0	30	30	4	125	
**EX 10**	**30±2**	**0.5**	**25**	**12.5**	**2**	**150**	**+++++**
EX 11	30±2	1.0	30	30	2	150	
EX 12	30±2	1.0	25	25	2	175	++
EX 13	30±2	1.5	20	30	3	175	
EX 14	30±2	0.5	20	10	2	200	+

Note. DOI: date of injection, Conc.: Concentration, Vol.: Volume, amt.: Amount, + denotes minimum and ++++ denotes maximum observed fluorescence. Each experiment (EX) was done in minimum three animals.

Table S2. Table showing comparison of occurrence of transgene positive pups in percentile, when different constructs were used as transgene.

**Transgene**	**Tg Positive Pups/Total Pups Born (in G1)**	**Instance of Tg Positive Pups**
Bucn2-IRES2-Egfp	12/17	70 %
Amh-IRES2-Egfp	8/25	32 %
Fetuin-A-ShRNA	16/36	44 %
**Total**	**78/36**	**46 %**

Table S3. Primer sequence used for genotyping of transgenic lines

**Si. No.**	**Constructs**	**Primer 5’ 3’**	**T_{anneal} (^{0}C)**	**Product Size (bp)**
2	**Amh-IRES2-Egfp**	**F:** AAGCCCTTTGAGACAGTCGC<br>**R:** ATATAGACAAACGCACACCG	62	295
4	**Bucsn2-IRES2-Egfp**	**F:** GAAACAATCTAGTCAATCCAAG<br>**R:** ATATAGACAAACGCACACCG	62	900
